# Artificial Intelligence-Based Semantic Internet of Things in a User-Centric Smart City

**DOI:** 10.3390/s18051341

**Published:** 2018-04-26

**Authors:** Kun Guo, Yueming Lu, Hui Gao, Ruohan Cao

**Affiliations:** Key Laboratory of Trustworthy Distributed Computing and Service, Ministry of Education, Beijing University of Posts and Telecommunications, Beijing 100876, China; guokun@bupt.edu.cn (K.G.); huigao@bupt.edu.cn (H.G.); caoruohan@bupt.edu.cn (R.C.)

**Keywords:** Internet of Things, smart city, smart home, artificial intelligence, semantic modeling

## Abstract

Smart city (SC) technologies can provide appropriate services according to citizens’ demands. One of the key enablers in a SC is the Internet of Things (IoT) technology, which enables a massive number of devices to connect with each other. However, these devices usually come from different manufacturers with different product standards, which confront interactive control problems. Moreover, these devices will produce large amounts of data, and efficiently analyzing these data for intelligent services. In this paper, we propose a novel artificial intelligence-based semantic IoT (AI-SIoT) hybrid service architecture to integrate heterogeneous IoT devices to support intelligent services. In particular, the proposed architecture is empowered by semantic and AI technologies, which enable flexible connections among heterogeneous devices. The AI technology can support very implement efficient data analysis and make accurate decisions on service provisions in various kinds. Furthermore, we also present several practical use cases of the proposed AI-SIoT architecture and the opportunities and challenges to implement the proposed AI-SIoT for future SCs are also discussed.

## 1. Introduction

As the future trend of city development, the smart city (SC) can provide convenient services for people [1]. The SC covers many domains, such as urban infrastructure, resident living environment, transportation management, medical treatment, shopping, security assurance, and so on. It is worth noting that the study of the SC starts with the smart home (SH), which is often treated as the basic unit of the SC. In the early days of SH, the concept of smart product is proposed and these products are often work without networking and inter-connection [2]. Recently, as the development of the Internet of Things (IoT), multiple products/components of SHs are connected to work in a collaborative fashion, and the SH can provide more meticulous services [3], for example, energy management [4], patient assistance [5], real-time product labelling [6] and subscribing [7].

**Figure 1:**
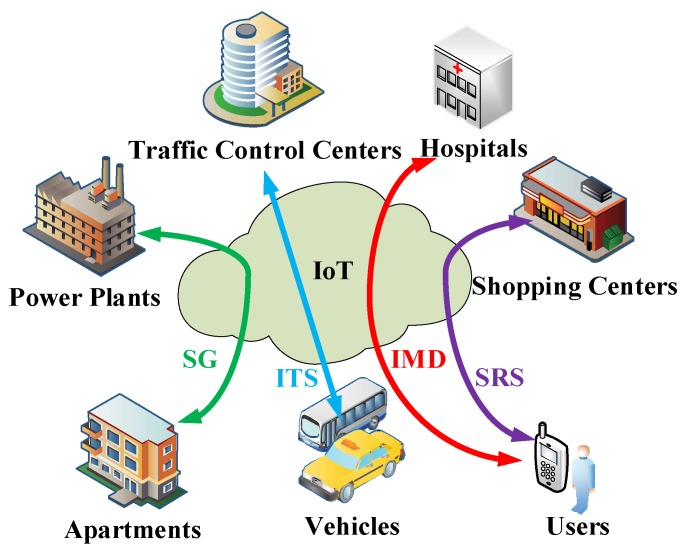
Smart city scenarios.

Today, a massive number of smart devices are joining the Internet, and the IoT technologies are in empowering various applications in the SC beyond the SH [8]. In addition to physical devices, some virtual objects are also included in SCs (e.g., properties of objects, data generated by devices and human characteristics). The SC may consist of various scenarios, as shown in Figure 1, including the Smart Grid (SG), Intelligent Transportation System (ITS), Intelligent Medical Diagnosis (IMD), Shopping Recommender System (SRS), and etc. To elaborate further, the SG can optimize the power supply to reduce the total energy consumption. The ITS can provide the best trip route for travelers. The IMD, on the other hand, can suggest reasonable medical plans according to the patient’s situation. Finally, the SRS can recommend appropriate products according to users’ demands. It is noted that these typical application scenarios in SC are based on the intelligent transmission and processing of a massive amount of data collected from various devices/objects in the SC. Moreover, the intelligent services that help users to make smarter decisions are increasingly popular, because the quality of experience (QoE) is increasingly important in SC applications [9]. However, the demand for intelligent services requires an extremely strong data processing capability, which motivates the artificial intelligence (AI) empowered system in the SH [10].

More specifically, AI uses various learning techniques to facilitate automatic resource provision and judicious decision making. Therefore, the SC empowered with AI is expected to be intelligent [11]. In academia, AI has been studied for over 60 years and more and more practical applications are emerging in recent years. One of the key applications is to make quick and optimal decisions according to real-time situations, and it has been demonstrated recently that AI can outperform human beings in many areas of interests, for example, the Google’s recent AI application Alpha Go [12]. In general, the AI application can be divided into two modules. The first and the most important one is the model-learning module, which is mainly responsible for effective data collection, data training and modeling. For example, the model learning module of the Alpha Go program should learn how to win the game. It must be trained by learning a large number of game situations/solutions in the stage of data collection and training. The corresponding solutions/strategies are summarized, and the corresponding learn-and-decide model is constructed in the modeling stage. On the other hand, another module is the predicting module, which is responsible for making actions to respond to the current situation. For example, the predicting module of Alpha GO can analyze the current game situation according to existing solution models, then, the best solution is implemented based on the analysis result.

Conventional AI applications are usually developed for specific application scenarios or objects, in addition, they require in-depth customization [13]. Therefore, conventional AI applications might be difficult to adapt to a general scenario that consists of multiple objects. However, many different types of objects coexist in SC and they are probably beyond the scope/capability of the traditional AI applications. To this end, new AI techniques should be developed to enable general applications. These objects access the Internet depending on different devices and functions that may follow different standards and patterns. Therefore, aiming to ensure that the AI solves the SC service problems through IoT technology efficiently, it is crucial and necessary to construct a platform that maps different behaviors of objects to a unified model. More specifically, the unified model can support the fusion of heterogeneous objects, and it eventually facilitates the AI processing of the system. In particular, the platform and unified model are supported by the semantic technology, which is able to describe the characteristics of objects for more efficient machine understanding and realize interoperability among multiple heterogeneous systems.

In this paper, an AI-based semantic IoT (AI-SIoT) hybrid service architecture is proposed in conjunction with the key technologies. The architecture is expected to address the aforementioned challenges, support heterogeneous devices, and find applications in practical scenarios. The way to embed AI into semantic IoT is described in detail and the implementing pattern of AI-SIoT is also explained. At last, we describe some typical use cases, which are based on our AI-SIoT service architecture.

## 2. Related Work

Recently, the study of SC architecture has attracted ample attention from both academia and industry. The main goal of the SC is to understand the requirements of users and provide appropriate services accordingly. The requirements of users can be acquired by analyzing user activities. For example, healthcare requirements can be discovered according to some special activities of patients, e.g., those who suffer from Alzheimer’s disease show a typical syndrome. Disease impairs people’s daily activities, and some novel SC applications should be developed to aid the patients. Dawadi et al. proposed a clinical assessment method according to the behavioral data in a smart home [14]. The method constructs an activity assessment model that evaluates the activities of daily living based on related clinician-provided cognitive assessment scores. The abnormal activity can be discovered, and the related diseases are predicted according to the evaluation scores. Similarly, Abdulsalam et al. proposed a Bayesian activity model based on the temporal energy consumption patterns of applications in smart homes [15]. Interestingly, the anomalous activity can be recognized by analyzing the energy usage changes, and the healthcare services can be provided accordingly. Jens et al. proposed a behavioral pattern discovering method based on the time and space factors in the smart home [16]. The associations between different behavioral patterns are considered, and the pattern transitions are modeled by the third order Markov chain. 

The safety requirements can also be addressed by analyzing the environmental data detected and collected from people’s activities, smart applications and building monitors. Huang et al. proposed an approach to support judicious decision for safety services based on the semantic ontology model in a wireless sensor network [17]. The approach is able to explore the target context and recognize risk factors through reasoning, and a series of ontology models are constructed.

In the aspect of intelligent service provisions, some IoT and SC architectures have been proposed in recent literature. Per et al. proposed an IoT architecture that integrates smart homes and smart cities through the Cloud-of-Things (CoT) [18]. The architecture updates data from IoT devices to the cloud, and the intelligent service is provided through the AI system. Bharti et al. proposed an intelligent resource inquisition framework with three layers, which are perception, discovery, and application [19]. The architecture can access the context information and provide services through a semantic match-making engine based on ontology models. Paula et al. proposed a simplified architecture that provided services through a hybrid data processing model, including historical data analysis and real-time analysis [20]. This architecture supports data ingestion, data retrieval and machine learning to determine the services to be provided. Charbel et al. proposed a semantic rule engine (SRE) for industrial gateways [21]. The SRE can handle semantic queries and infer the required services. 

In the aspect of mobile crowd sensing for SC, Guo et al. [22,23,24] presented a review on Mobile Crowd Sensing (MCS) and proposed a novel framework of Mobile Crowd Sensing and Computing (MCSC), which allow mobile users share their personal data. The framework can realize explicit/implicit sensing and heterogeneous, cross-space data mining. Guo et al. also presented the fusion of human and machine intelligence to select the proper human to meet the specific needs. Zappatore et al. [25] proposed a MCS platform in SC to sense users’ activities and opinions, give suggestions about the noise abatement interventions to city managers and provide low-cost, large-scale monitoring tool for potential noise pollution risks. Alvear et al. [1] proposed an analysis of candidate technologies for crowd sensing architectures and presented a design of an off-the-shelf mobile environmental sensor, which can meet the air quality monitoring requirements. Longo et al. [26] constructed a platform named Urban Mobile Sensing as a Service to monitoring noise, air, electromagnetic fields. The platform is based on MCS paradigm and it can collect data from SC to improve citizens’ quality of life and help city managers to make decisions. Corradi et al. [27] presented a MCS platform and it leverages communities to increase people involvement in MCS campaigns by using k-CLIQUE algorithm. Habibzadeh et al. analyzed the smart city applications’ usage of distributed sensor network and presented SC sensing systems, which cover dedicated and non-dedicated sensors [28]. The dedicated sensors are purposed for specific applications and the non-dedicated sensors is formed by connected smart devices. Panichpapiboon et al. proposed a mobile sensing approach for traffic density estimation [29]. The approach uses vehicles as mobile sensors and the traffic data can be collected by users’ smartphones. Cortellazzi et al. presented an extension of the general-purpose ParticipAct platform based MCS [30]. The platform considers the mobile application, the website, the GIS map to help the people with impaired mobility to share knowledge between them. 

In the aspect of IoT-based applications for SC, Hsu et al. proposed a RFID-based indoor tracking system for elderly people living alone [31]. The system collects the signal strength data of RFID reader and coordinates with wireless sensor node of a three-axis accelerometer to compute the users’ locations. Purri et al. described the IoT-based healthcare system in hospitals, and the system can monitor patients using sensors and allows objects to be detected and controlled remotely [32]. Martinez et al. developed an information tracking system based on RFID technology for patients and evaluated the system from some key indicators, such as suitability, cost, efficiency, usability, medicine tracking, patients tracking and safety [33]. Catarinucci et al. proposed a context-aware smart infrastructure and related smart applications based on Ultra High Frequency (UHF) RFID technology, which proposes new RFID tags having the capability to transmit data measured by sensors [34]. Amendola et al. analyzed the current RFID technology-based applications for IoT healthcare, such as body-centric systems (detecting users’ gestures) and environment monitor systems (detecting temperature, humidity, and other gases) [35]. Talari et al. analyzed the IoT Technologies for SC, such as RFID, Near Field Communication (NFC), Low Rate Wireless Personal Area Network (LWPAN), Wireless Sensor Networks (WSNs), Dash7, 3G and Long Term Evolution (LTE), etc. [36]. They also proposed IoT potential applications in some SC field, such as smart cities and communities, smart homes and buildings, responsive customers, smart energy and smart grids. Esposito et al. proposed a context-aware framework for alert generation by using ontological context representation, which can realize rule-based reasoning [37]. Pang et al. proposed a pervasive and preventive healthcare solution for medication noncompliance and daily monitoring [38]. The solution implements the RFID-based intelligent package and multi-core computing technologies. Majumder et al. analyzed the current research and development on wearable systems for health monitoring, such as cardiovascular monitoring system, activity monitoring system, body temperature monitoring system, galvanic skin response monitoring system, blood oxygen saturation monitoring systems, etc. [39]. Yang et al. presented a IoT-based intelligent home-centric healthcare platform, which collects data from the smart sensors attached to human body and update the data to the cloud for the daily medication management [40]. 

In the aspect of edge-computing services for SC, Song et al. built a smart collaborative caching scheme in IoT through high-level Information Centric Networking (ICN) principles. Through the analysis of typical scenarios, it is concluded that the scheme optimizes the total packet number and average transmission latency [41]. Hou et al. proposed a green survivable virtual network embedding (GSVNE) for the collaborative edge computing in SC to guarantee the network survivability [42]. In the method, the number and geographical locations of backup Edge devices are determined by resource-division methods based on heuristic strategies and the GSVNE will ensure the maximal sharing degree of backup resource. Higashino et al. [43] mentioned that Information Communication technology (ICT) for disaster mitigation and SC research problems that are expected to develop in the next ten years are enumerated, so as to build a safe and intelligent city against disasters. The development of Internet, smartphones, IoT devices has brought great changes to the collection and distribution of disaster information, however, there is still room for development if we combine multiple technologies to support disaster. Sapienza et al proposed a SC architectural model with mobile edge computing and fog computing exploits Mobile Edge Computing (MEC) concept [44]. The approach distributes the computational load onto the network equipment and the program that leveraging nodes to deploy service for SC improves the user experience. Santos et al. proposed a fog-computing framework that enables 5G enabled SC with autonomous management and orchestration functions [45]. The framework fully integrated fog node management system and Open Shortest Path First (OSPF) routing protocol applied to exchange information between fog nodes. Evaluation results show that network bandwidth usage and latency reduced obviously. Reference [46] proposed a follow-me cloud-cloudlet approach in fog-computing-based radio access networks for SC which can reduce the latency of data transmission in SC.

It is noted that the approaches discussed above do not integrate AI and semantic models based on natural language. Moreover, the ontology models are limited by the formulation structure and are therefore lack sufficient flexibility towards the AI system. Motivated by these observations, in this paper, we proposed the AI-SIoT to address the challenges confronted by the existing systems.

## 3. AI-SIoT Architecture

**Figure 2:**
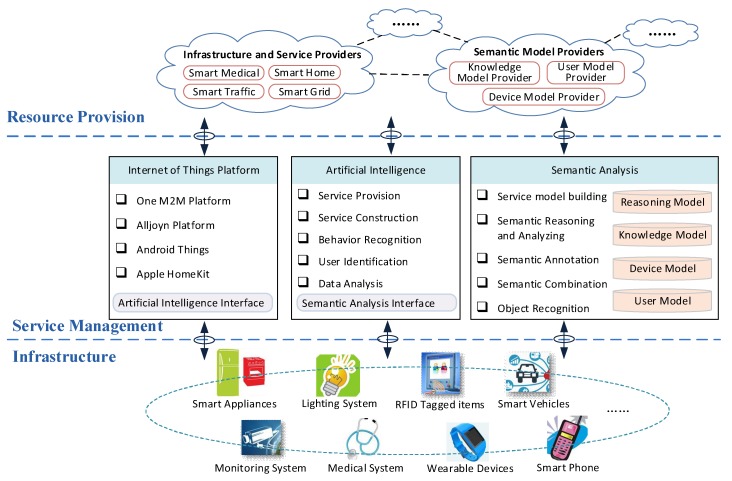
AI-SIoT architecture.

The overall AI-SIoT architecture is illustrated in Figure 2 and it is composed of three layers: the infrastructure layer, the service management layer and the resource provision layer.

### 3.1. Infrastructure Layer

The infrastructure layer includes all kinds of smart devices in the SC IoT, such as smart appliances in SHs, smart lighting systems, RFID tagged items, smart vehicles, smart monitoring systems, smart medical systems, wearable devices, smartphones, and more. The IoT-accessed smart device is the basis to construct the IoT. The smart device can be divided into three different types, including sensors, actuators, and hybrid devices. Sensors are mainly used for sensing the environment, and can be classified as the temperature sensors, humidity sensors, light sensors, cameras, smart bands, RFID readers and etc. In a simple sensor system, the actuator takes actions when it receives a command. In a more advanced sensor system, hybrid devices equipped with sensing and actuating modules and perform more complex functions. For example, in our living and working environments, most of the devices are hybrid devices including televisions, refrigerators, smartphones and smart watches. Because all smart devices are designed to serve human beings, a large amount of data can be produced during the interaction among users. The data can also be recorded and uploaded to the service management layer for further analysis.

### 3.2. Service Management Layer

The service management layer is mainly responsible for device management, data analysis and service provision. It is deployed in the Cloud and the service area. It associates the user with the Cloud. There are three important uncoupled modules: the IoT platform, the AI module and the semantic analysis module.

#### 3.2.1. IoT Platform

The IoT platform is the accessible entrance of the IoT for the smart devices. Examples include but are not limited to the oneM2M platform, the Alljoyn platform, the Google Android Things and the Apple HomeKit. The oneM2M is an international standardization organization in the field of IoT and the oneM2M platform provides a universal resource framework, which allows oneM2M devices to register to the resource pool. Alljoyn is an open-source software framework originally developed by Qualcomm and the Alljoyn platform provides a complete and comprehensive P2P solution, which does not depend on a specific communication protocol. The Google Android Things is an IoT operation system and it can speed up the development of IoT devices based on Android system and related applications. The Apple HomeKit focuses on the SH field and the Intelligent devices in SH can be managed by Apple’s virtual assistant Siri. These IoT platforms control the accessed devices and collect device data. The IoT platform is usually divided into two associated submodules. One submodule is deployed around the smart devices to ensure their access to the IoT, and it also provides the access points. As a submodule of IoT platform, a smart gate can help the smart devices to access the IoT, and it is usually seen as the bridge between the infrastructure layer and the service management layer. The other submodule is deployed in the Cloud, and it provides the remote management, data analysis, and other extended services. In our AI-SIoT architecture, an AI interface is constructed to link the IoT platform and the AI module. All platforms can leverage the AI interface to access the AI module for data analysis. Although the IoT standards tend to be unified, a variety of different standards and platforms will still coexist for a relatively long time in the years to come. To this end, it is necessary to provide the same interfaces for the intelligent interactions between different platforms.

#### 3.2.2. AI Module

The AI module contains five submodules: data analysis, user identification, behavior recognition, service construction and service provision. In the AI module, submodules can leverage the semantic analysis interface to implement semantic analysis. The IoT platform uploads the data to the AI module through AI interface and the data analysis submodule receives and analyzes the collected data from the infrastructure. In the data analysis submodule, the data characteristics are abstracted and the data changing patterns are mined via on time series analysis for behavioral modeling.

The data analysis technology is the basis towards AI. In the IoT-based SC scenarios, there is abundant of user data produced every day. The user data represents the user’s daily life or a period of life pattern [47]. The data analysis technology can obtain the extended data that represents the model of a user’s life pattern. There are four major steps for data analysis, including data collecting, data training, data modeling and data predicting. In the aspects of data training and data modeling, there are many useful algorithms, such as the Hidden Markov Model (HMM) [48], Naive Bayesian (NB) [15], Support Vector Machine (SVM) [3], Decision Tree (DT) [14], Artificial Neural Network (ANN) [47], Deep Learning (DL) [9], and so on. Forkan et al. used a HMM based approach for detecting abnormalities in daily activities [48]. In the approach, a statistical model is constructed for understanding irregularities in daily routines, a disease prediction model is described to measure the trends of physiological states and a fuzzy rule-based model is used to discover the true anomaly. Bisio et al. constructed a smartphone-centric Ambient Assisted Living platform to monitor patients suffering from physical and mental limitations [3]. An activity recognition method based SVM is proposed to evaluating user behavior though analyzing users’ information about audio, localization, and movement. Bourobou et al. proposed a K-pattern clustering algorithm to acquire fine-grained temporal relations and construct user activity models [47]. The ANN is then used to recognize and predict users’ next activities based on the existing activity models, which are based on historical data. He et al. suggested to emphasize users’ QoE requirements to improve the big data services, such as smart grid, smart healthcare and smart tourism [9]. A deep-learning based greedy algorithm is proposed to acquire users’ QoE requirements and enhance intelligent services.

In particular, the HMM is usually implemented for sensing the intentions of users according to the known behaviors. For example, a behavior that the user picks up a cup may indicate he/she wants to drink. NB is used for mining the behavior sequence of a user. For example, watching TV may contain a behavioral sequence such as going into the living room, turning on the TV set and sitting on the sofa. SVM, DT, ANN and DL can leverage the collected characteristic data to construct the behavioral prediction model. 

In addition, there are some hybrid modeling methods for behavioral modeling. Chen et al. presented an activity model based on knowledge-driven and data-driven approaches [5]. The model can be used to recognize users’ activities in the initial stage based on the existing ontology-based activity models. With the increasing activity data, the new activity patterns will also be discovered through data mining. Cook et al. proposed a pattern discovery method of complex activity based on predefined activity models [49]. The method considers the relationships between the predefined activity models and unlabeled activity data to construct new activity patterns. Similarity, Azkune et al. presented an activity clustering method based on initial simple activity models, which are constructed through knowledge engineering [50]. These knowledge-driven activity models are fine-grained and they will be improved to be complete and specialized activity models through pattern mining.

**Figure 3:**
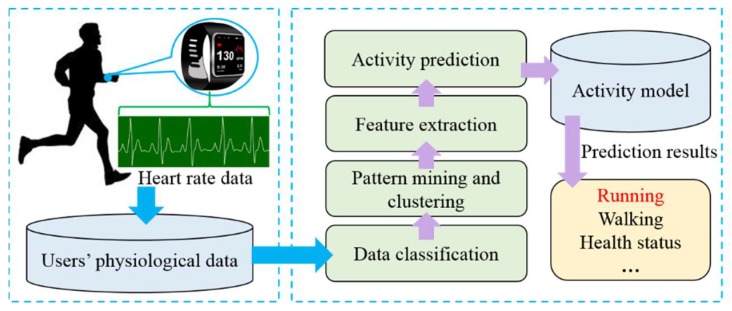
Running predicting based on heart rate.

These approaches are usually applied to predict the activities of users. For example, the heart rate is monitored and the rate changing pattern can indicate the user’s activity as shown in Figure 3.

Generally speaking, smartphones report users’ locations timely and periodically. The location data can be seen as a trigger event for a location-based automation service or as a logical reference for the recommendation service. In addition to the location data, the users’ behaviors and actions are reported. These data are the important basis to construct the behavioral model of users that can represent the behavioral patterns inferred through machine learning techniques. A behavioral model usually contains time, locations, objects and contents. The behavioral content represents the interaction with the current target object.

The behavioral modeling can refer to human beings and smart devices. There are two types of data in the modeling: registration data and service data. The registration data is produced when the smart devices are registered in the IoT platform. It may contain the basic information about the registered devices, such as the name, type, device ID, manufacturer, and more. The service data is produced in a timely manner by the smart devices, and it can represent the device’s working status. The working state can be the on/off status, or some other functional states. In addition, the function data can be the sensed environmental parameters and the calculated working durations and etc.

In the user identification submodule, the user identity can be detected and confirmed. In general, the smartphone, wearable devices or other personal devices can serve as the entrance to the SC via user logging. Through the user interfaces, these devices can perform multiple functions, such as locating users, controlling other devices, recording user characteristics, and more. The user characteristics can be used to describe users and facilitate the provisions of appropriate services. The submodule is empowered by the semantic analysis through the corresponding interface. The semantic analysis module can acquire detailed user information in various aspects. In the behavioral recognition submodule, the user behavior and device behavior are recognized and confirmed through the supports from the data analysis submodule and the semantic analysis module. The user behavior represents a series of user activities for a certain purpose, and the combinations and sequences of these activities are regular. The main objective of the behavior recognition submodule is discovering the behavioral patterns and constructing the behavioral model. For example, a user often puts milk in coffee with no sugar. The milk and coffee can be the key elements for the behavioral pattern of the drinking coffee and sugar may be included in another behavioral pattern. The user behavior recognition can provide assistance in user’s daily living [51]. Similarly, the device behavior represents that one device or some devices implement a series of activities within a certain period of time. These activities of devices are implemented through manual operations or automatic operations. The automatic operation generally results from some trigger events. For example, an air conditioner is set to start cooling when the indoor temperature is above 30 °C. The indoor temperature is one example of the trigger event. The relationship between the two different behaviors, such as opening the door and turning on the air conditioner, is that the user behavior interacts with the devices, but the device behavior interacts with the environmental parameters or controllable events. Changing the environmental parameters or some events is the main purpose of users that are using devices. The user behavior can stimulate the corresponding device behavior. For example, people control the air conditioner, and the air conditioner can reduce the temperature. Some different device behaviors may result in the same results. Therefore, they may be interchangeable in some special situations. For example, in the summer, air conditioners and electric fans can both reduce the temperature. The air conditioner may be the preference, but when it is broken, the electric fan could be the next choice. In addition, the results of device behavior can be recorded to analyze the user’s intentions. The automation services can therefore be constructed and provided to users according to the user’s intentions. The service construction submodule is mainly in charge of the preparing of the available services, including the original services and the learned services. The service provision submodule can offer the prepared services to the users.

#### 3.2.3. Semantic Analysis Module

**Figure 4:**
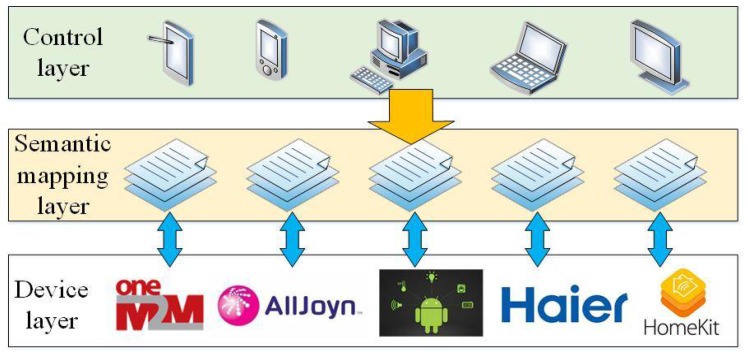
The semantic mapping layer for heterogeneous IoT platforms.

The semantic analysis (SA) module provides basic information of semantic analysis for user identification, behavior recognition and service construction in the AI module. Semantic technology can construct a semantic mapping layer through constructing various semantic models, including the device model, user model, knowledge model and reasoning model [52,53]. The semantic mapping layer enables different IoT platforms to hide their heterogeneity, which is shown in Figure 4.

There are many coexisting IoT platforms for device access, such as the Haier U-home platform, Apple HomeKit, Google Android Things, the Alljoyn platform, the oneM2M platform, and so on. Specifically, OneM2M is committed to establishing global standards for the IoT [54]. It refers to the areas of health care, energy management, entertainment, security and etc. However, the truly global standard for smart device management in the IoT is yet to come. Smart devices from different providers follow their own standards, including device descriptions, functional instructions, control interfaces. Different standards lead to inflexible and expensive IoT configurations, and they slow down the development of unified IoT services. Therefore, it is necessary to make these different IoT platforms access the same AI module to ensure the interaction among different IoT platforms. Then, people can flexibly enjoy services from different IoT platforms for the same intention. In addition, semantic technologies enable devices to understand human intentions. Semantic technologies describe people’s living environments based on natural language processing, which is also the key to the machine understanding. In traditional intelligent service solutions, the voice control technology is implemented and people can acquire services by giving voice commands and instructions. However, these commands and instructions are only segmented and matched with an inherent instruction library. The traditional intelligent service is not based on machine understanding, and it is not intelligent enough. Semantic technology requires everything to be associated to understand users’ activities and predict users’ intentions. Semantic computing can be implemented through semantic matching [21,55,56], including association computing and similarity computing. Association computing determines a series of services that people need. Similarity computing determines the alternative services for people’s same intention. For example, when one service is not available, another service can achieve the same effect.

**Figure 5:**
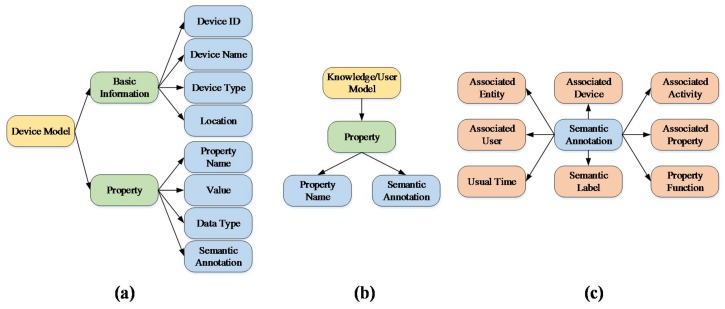
Semantic model including (**a**) device model; (**b**) semantic annotation; (**c**) knowledge and user model.

There are five submodules in the semantic analysis module. When a new smart device is registered to the IoT platform, the registration data can be leveraged to match the corresponding semantic models in the object recognition submodule. This submodule connects the Cloud, acquires the semantic model of the new device from the device model provider, and constructs an instance according to the semantic model. The instances of the new devices are stored in the device model database. Our proposed semantic models (including device model and knowledge/user model) are illustrated in Figure 5. Specifically, Figure 5a shows that the device model contains two categories of information branches. First, the basic information mainly contains the device ID, device name, device type and location. Second, the properties mainly contain the property name, value, data type and semantic annotation. The semantic annotation is the key element to construct the associations among devices, which is described in Figure 5c.

The Semantic Annotation records the association information of various devices, including associated entities, associated devices, associated activities and etc. These contents are usually used for inferences, because they describe the device properties in detail from various aspects. For example, the air conditioner has the property of cooling, which can reduce the environment temperature. Another property of an air conditioner is temperature detection, which detects the environment’s real-time temperature. Meanwhile, the semantic annotation of the cooling records the associated property as temperature detection, and the property function has defined the rule to trigger the cooling function when the temperature is above a certain value. Then, the whole process can work through the cooperation of cooling and temperature detection. The recorded information of the semantic annotation will always be updated with the data of users’ activities and other changing knowledge.

Similarly, the user models and knowledge models are acquired from corresponding providers, which are shown in Figure 5b. User models mainly represent the user characteristics, preferences and requirements. Knowledge models represent the general methods, instructions, common senses, and so on. For example, there is a new cooking method using the intelligent oven on the Internet. The knowledge information can be collected and recorded in the semantic annotation of the corresponding device if the device type is matched. Then, the new method is recommended to the user when the user wants to use the oven for cooking. 

The semantic combination submodule associates one semantic annotation with another by referring to the device model and knowledge model. The association process is supported by the semantic association computing. Each property of the device may have multiple semantic annotations to show what this property can do. When the association value between two semantic annotations from different devices is above the given threshold through the association computing, the devices can be associated, and their corresponding properties can constitute a new virtual device (VD). The VD is defined as a set of device properties for satisfying users’ requirements, and it is constructed as a reasoning model. These properties belong to different devices, and they form an implementing sequence. For example, a user wants to cook fried eggs. He/she needs to take some eggs from the refrigerator, take out the pan from the cupboard, and turn on the gas cooker. The refrigerator, the pan and the gas cooker constitute a new VD for cooking. The semantic annotation submodule provides additional property annotations based on the existing ones to construct related semantic models. The additional semantic annotations are dynamic and can be continually rewritten according to the user’s behaviors and device’s behaviors. The semantic reasoning and analysis submodules can construct reasoning models according to the associations between the semantic models, including user models, device models and knowledge models. The semantic annotations are the basis of semantic reasoning. The service model is built in the service model building submodule according to the analysis results and it represents introductions of what and how the user should behave. The service model building submodule is called by the service construction submodule in AI through the semantic analysis interface.

**Figure 6:**
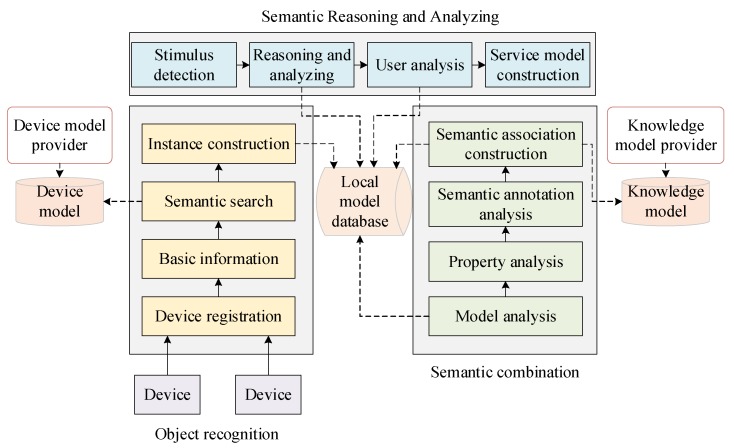
Semantic analysis process.

The whole process of semantic analysis is illustrated in Figure 6. When devices access, they will register to the platform and their basic information will be used for semantic model search. Then their instances will be stored in the local model database. The semantic combination will analyze these instances and implement semantic annotation and semantic association construction according to the knowledge models. When the service requirements are detected, the semantic reasoning and analyzing will be called for finding related services. At last, the users’ model will be analyzed and the appropriate services will be constructed.

### 3.3. Resource Provision Layer

The resource provision layer mainly contains resource providers in the AI-SIoT. Infrastructure and services providers can provide services for smart homes, smart traffic, smart grids, smart medical and etc. Semantic model providers construct and provide semantic models in various fields for the semantic analysis module, such as the device model provider, the knowledge model provider and the user model provider. The device model providers usually cooperate with the device manufacturer to design corresponding semantic models. The knowledge model provider should collect the various latest knowledge data. There are two issues about the semantic model. One issue is how to make the different devices understand each other, and another one is that how to mine the effective associations between semantic models. The two issues determine the quality of the model. In addition to the two basic resource providers, there are a variety of providers in other extended fields in the Cloud, such as the social field, the education field, the business field, and more. Moreover, these resource providers provide the basic resources for AI and semantic analysis in service management layer. Meanwhile, the AI and semantic analysis provide data analysis and reasoning for the resource providers to understand the users’ intentions. Consequently, the resource providers can provide services with a high QoE.

## 4. Use Cases

In this section, we proposed three use cases, namely the basic intelligent services, the service based on associations of semantic models, and the semantic annotation and reasoning. In addition, we develop a prototype and analyze in details for the use case of semantic annotation and reasoning.

### 4.1. Basic Intelligent Services

**Figure 7:**
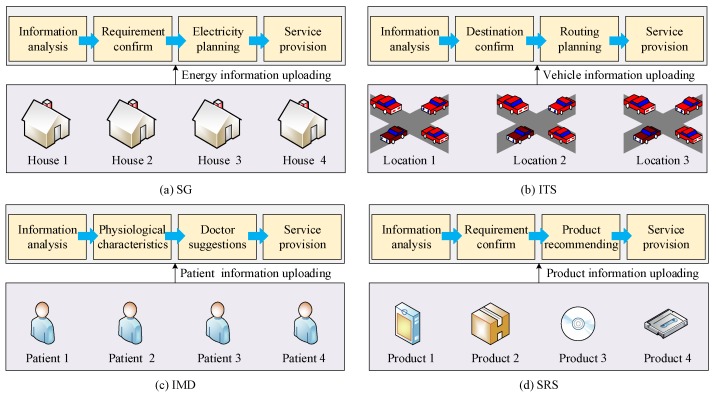
Processes of basic intelligent services.

In the SG scenario, which is shown in Figure 7a, the information about energy can be treated as a property of a device. The properties of all devices that are used, can be integrated, scheduled and optimized. The users’ requirements for electricity are analyzed to make a reasonable plan. The objective is that the data value of energy consumption can be reduced. Then the energy service will be implemented. In the ITS scenario, which is shown in Figure 7b, moving vehicles can be seen as objects accessed in the Internet of Vehicles (IoV), which is one special type of IoT. The users’ destinations, location, speed and direction of these vehicles are uploaded to the management terminal server, and the reasonable scheduling schemes (vehicles’ routings) are distributed to every vehicle. Then, a better traffic environment can be guaranteed. In the IMD scenario, which is shown in Figure 7c, patients can record their own health-related data through a wearable device or other measuring equipment. The wearable device can measure some real-time data, such as the heart rate, exercise data and sleep-activity status. The other non-wearable device can periodically measure some relatively stable data, such as body weight, blood pressure, blood sugar and blood fat. In addition, patients can also take some necessary pictures and upload these pictures to the medical system through the Internet. The patients’ information represents the physiological characteristics, which can be used for disease recognition. The doctors can implement remote diagnosis for their patients according to the health-related data. In the SRS scenario, which is shown in Figure 7d, all the products in the supermarket are labeled by the RFID tags on the products. The product shelves are equipped with RFID readers. The RFID readers record the basic information and location of these products. When people go to the supermarket, their shopping habits are recorded and their requirements will be confirmed. In the management system of the supermarket, the recorded data will be analyzed to predict what customers need. Then, some specific products may be recommended to customers, and customers are guided to the corresponding product shelves by a robot. In addition to the SRS, there are other multiple-recommender systems. For example, diet recommendations can promote the appropriate food for a patient according to his/her health-related data. Known people’s preferences and characteristics, entertainment recommendations can introduce an appropriate place for people to relax.

### 4.2. Service Based on Associations of Semantic Models

**Figure 8:**
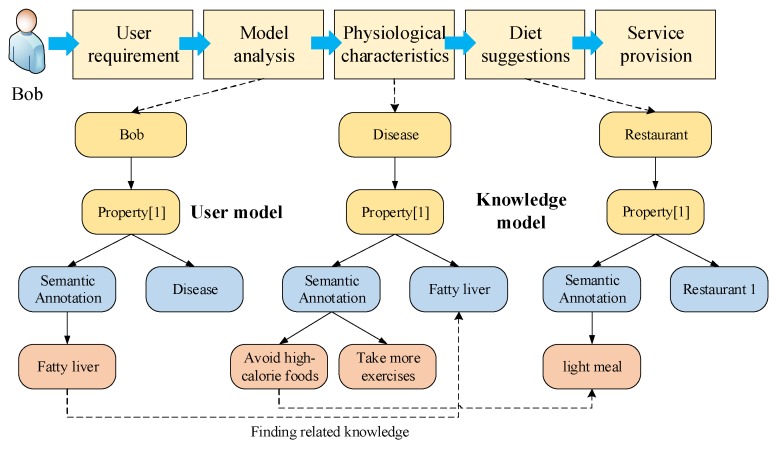
Processes of semantic associations construction.

Let us start with a motivating example, which is shown in Figure 8. A user Bob searches his favorite restaurants through a smartphone and the activity represents the user’s requirement. However, Bob has been diagnosed with fatty liver according to user model analysis. The fatty liver is written in the user model as a semantic annotation. As common sense, there is a strong correlation between fatty liver and food. There are two knowledge models need to be referred in the semantic reasoning. One is that people with fatty liver should avoid high-calorie foods. Another one is that a restaurant is a place where various kinds of foods are provided. Therefore, once the behavior of searching restaurants is recognized, the reasoning result is obtained through the semantic analysis based on some semantic models. The semantic reasoning and analysis submodule learns that Bob’s physiological characteristics will be analyzed and he should eat food with low salt and fat, and the total calories need to be limited according to the disease model. Then, a suitable restaurant will be recommended to Bob as the diet suggestions. When Bob finishes the dinner, he should do some exercises. The associations between the semantic models are integrated, and then a reasoning model is constructed and stored in the semantic analysis module. After that, the service model is constructed and the appropriate foods are displayed to Bob in conjunction with the advice to exercise after dinner.

### 4.3. Semantic Annotation and Reasoning

**Figure 9:**
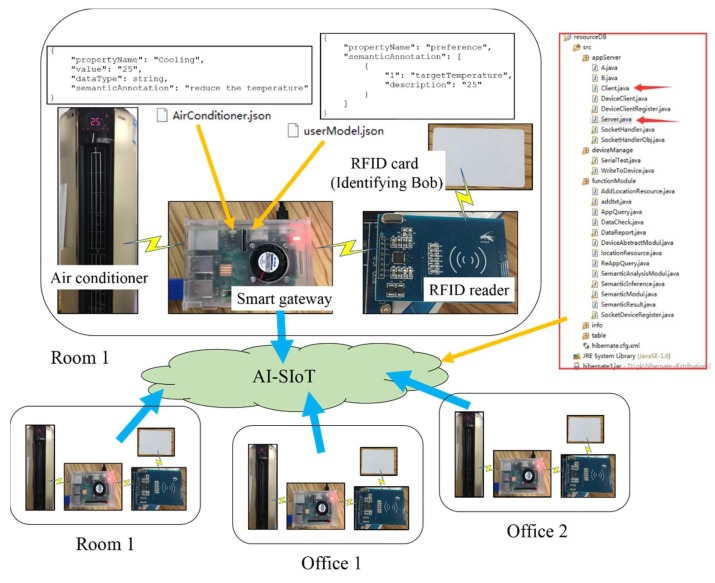
Prototype system of temperature control.

**Figure 10:**
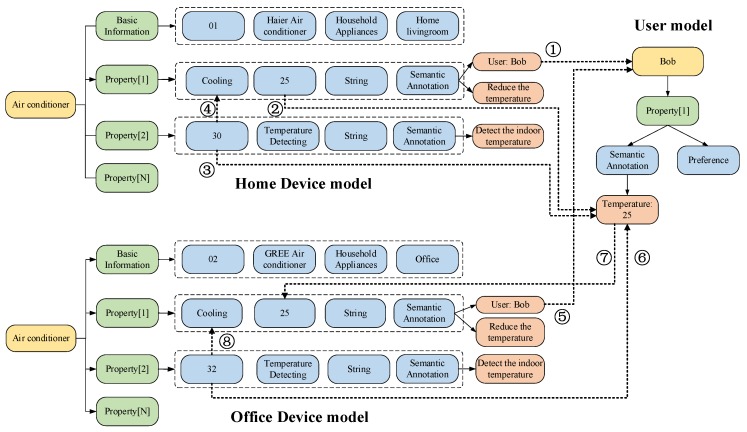
Instance of semantic annotation and reasoning.

In this use case, we perform detailed experimental analysis. When Bob is at home, he usually turns the cooling temperature of the air conditioner to 25 °C. Then, the temperature value would be written into the preference of the user model. When Bob enters the room again, the air conditioner can configure the user’s preferred temperature. When the user goes to his office, if the air conditioner of the office is in the AI-SIoT, the temperature can be set to 25 °C according to the shared user model. The prototype system is shown in Figure 9, where the smart gateway (oneM2M platform for devices) connects to the AI-SIoT and it manages the instances of the semantic models. The oneM2M platform is developed by Java. In addition, we have developed the server of oneM2M platform and the smart gateway will connect to the server. The RFID card identifies the user as Bob. The RFID reader is installed in the access control system of the room. The user uses the RFID card to enter the room. The air conditioner is developed by C++ in the control system, and it can detect and control the indoor temperature. The RFID reader and the air conditioner connect to the smart gateway through WiFi. All semantic models in our prototype are developed by Json. There are four similar rooms and offices connecting to the AI-SIoT. These rooms and offices are all about 15 square meters and we have put the same air conditioner, smart gateway and RFID readers in these rooms for the following experiments. User Bob can access each of rooms using the RFID card. The whole process of the semantic annotation and reasoning instance is based on the device model and the user model, which it is shown in Figure 10.

All data produced by the devices on the IoT platform will be analyzed by the AI module and SA module. In the step 1, the user identification submodule (in AI module) associates Bob’s user model through object recognition submodule (in SA module) when Bob’s identity information is sensed at home. In the step 2, the air conditioner (home) operations and related temperature value is recorded in Bob’s user model through the data analysis submodule (in AI module), behavior recognition submodule (in AI module) and the semantic annotation submodule (in SA module). In the steps 3 and 4, the semantic reasoning and analyzing submodule (in SA module) confirms the user’s intention (reduce the temperature). Then, when the Bob goes to his office, Bob’s identity information is sensed in the step 5 through the user identification submodule (in AI module) and object recognition submodule (in SA module). In the step 6, the semantic reasoning and analyzing submodule (in SA module) analyze the current temperature according to Bob’s user model. In the step 7, the service model building submodule (in SA module) confirm the target service content and the service construction submodule (in AI module) confirm the details about the air conditioner’s (in office) operations (services). In the step 8, the service provision submodule (in AI module) provides the corresponding services through the IoT platform.

In some situations, the same cooling temperature of the air conditioner cannot bring the same experience to users in different environments. For example, if Bob is in a computer laboratory rather than an ordinary room in Bob’s home, desktop computers and blade servers generate much heat and raise the laboratory temperature. In the room with no other heating devices, the user’s preferred temperature can be controlled by the air conditioner. However, in Bob’s situation, the original preference of the user model cannot be considered as the temperature setting reference. Instead, the body’s perceived temperature should be considered in the user model as a semantic annotation. Bob’s perceived temperature is estimated according to the user environment. For example, it may be estimated at home with no heating devices. However, in the laboratory, it is necessary to consider the impacts from the working computers, servers and laboratory area to estimate the raised temperature’s amplitude. These impacts’ value can be acquired by the sensors attached to the locations, where people always stay. These sensors will upload the temperature information to the smart gate way for further analysis. Then, the appropriate cooling temperature can be set by comparison with the results at Bob’s home. If there is more than one person in the laboratory, the average body perceived temperature could be used to describe all users’ preferences. When one of them has got a cold, the disease would be incorporated into the user model as a semantic annotation. The suitable temperature of the sick person would be given priority.

**Figure 11:**
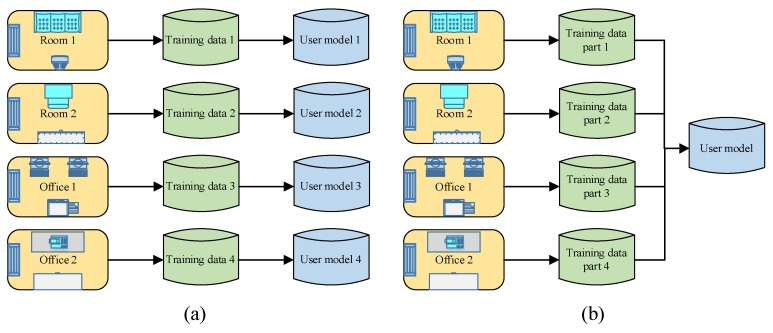
The construction of user model (**a**) Independent model (**b**) Shared model.

In addition, we analyze the effects on users’ QoE with the independent user model and the shared user model, which are shown in Figure 11. The independent model (IM) only contains the information of one user activity area, but the shared model (SM) can contain the user information of all the related activity areas.

**Figure 12:**
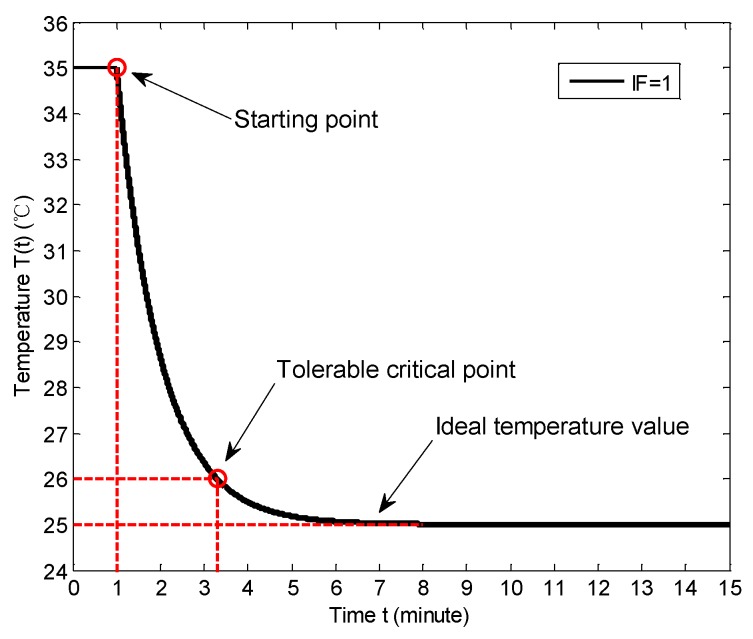
The indoor temperature variation when cooling.

**Figure 13:**
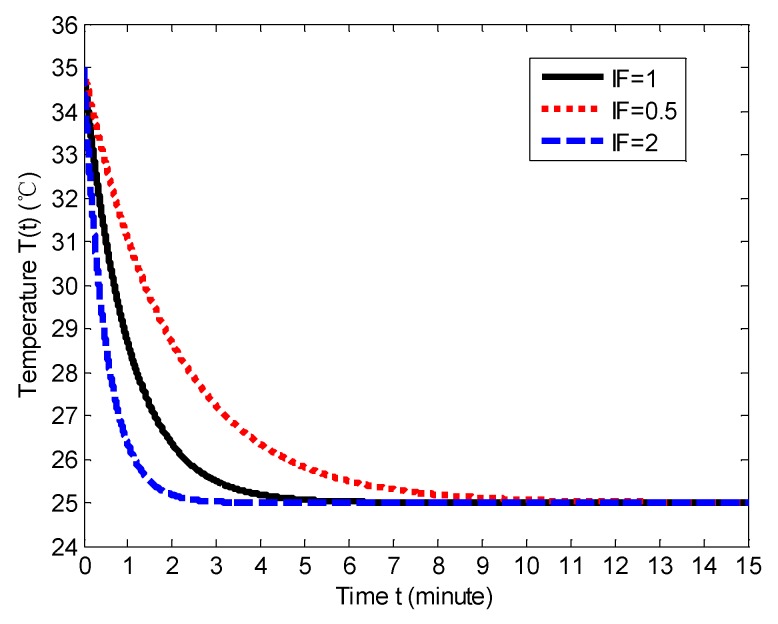
The indoor temperature variation with different IF value.

The model of user’s QoE can be constructed according to the user’s feelings, e.g., the indoor temperature is hot or cold [57]. According to our measured data, we can construct the temperature model in a room as:

(1) $$T(t)=(T_{0}-T_{c})\exp\left(-\frac{\alpha \cdot W}{\beta \cdot S}\times t\right)+T_{c}$$

where *T*_0_ is the current indoor temperature, *T*_c_ is the cooling set temperature, *W* represents the wind speed of air conditioner, *S* represents the floor space, the *α* and *β* are the tuning parameters, and *t* represents the time. The item $\frac{\alpha \cdot W}{\beta \cdot S}$ is the impact factor (IF). When IF = 1, the temperature variation curve is shown in Figure 12. The starting point represents the beginning of cooling and the indoor temperature is 35 °C before the starting point. The tolerable critical point represents the time when the temperature is in the acceptable range, which is defined as *T*_c_ ± 1 °C. In this paper we set *T*_c_ as 25 °C, which is the ideal temperature value. The tolerable temperature is set as 26 °C. Figure 13 shows the respective temperature variation curves with IF = 0.5, 1 and 2, respectively, when the cooling is on. We can note that when the IF increases (i.e., the *W* increases and the *S* decreases), the cooling effect becomes more prominent.

According to the temperature model, we can further establish the QoE model. The QoE represents the satisfaction with the service. Therefore, the differences between the current indoor temperature and the user’s tolerable temperature can indicate the user’s QoE. Then, we can define $f(t)=T(t)-(T_{c}+1)$, and the QoE model is defined as follows:

(2) $$Q=1-\frac{(T_{0}-T_{c}-1)\cdot t_{0}+\int_{0}^{t_{a}}f(t)dt}{\left(T_{0}-T_{c}-1)\cdot (t_{0}+t_{a}\right)}$$

where *t*_0_ represents the time of the starting point and *t*_a_ represents the time of the tolerable critical point. Equation (2) contains the duration before the starting point because of the user’s manual operation time. When the user model is finished, the QoE model can be defined as:

(3) $$q=1-\frac{\int_{0}^{t_{a}}f(t)dt}{(T_{0}-T_{c}-1)\cdot t_{a}}$$

We measure the QoE in four different rooms with different user models, and the detailed measurement parameters are shown in Table 1. T represents the initial temperature and the status represents whether the user triggers the air conditioner. In our experiments, we assume that the user model about the temperature preference should be confirmed, when the trigger frequency of the air conditioner is more than 3 (training critical value). The measurement results are shown in Table 2, which shows that the SM performs better than IM in the initial stage, especially for the rooms not frequently visited by the users.

**Figure 14:**
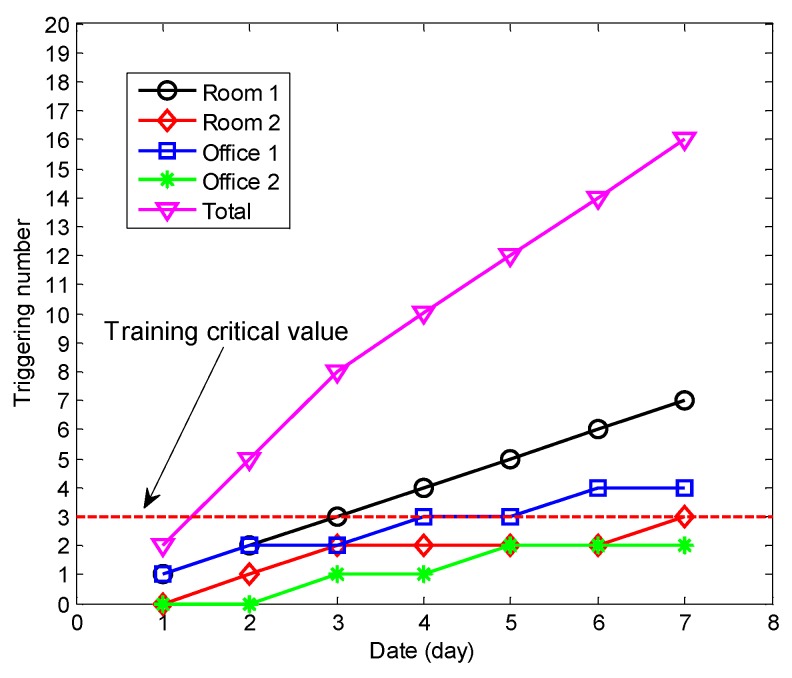
The triggering number of cooling in the four rooms.

**Figure 15:**
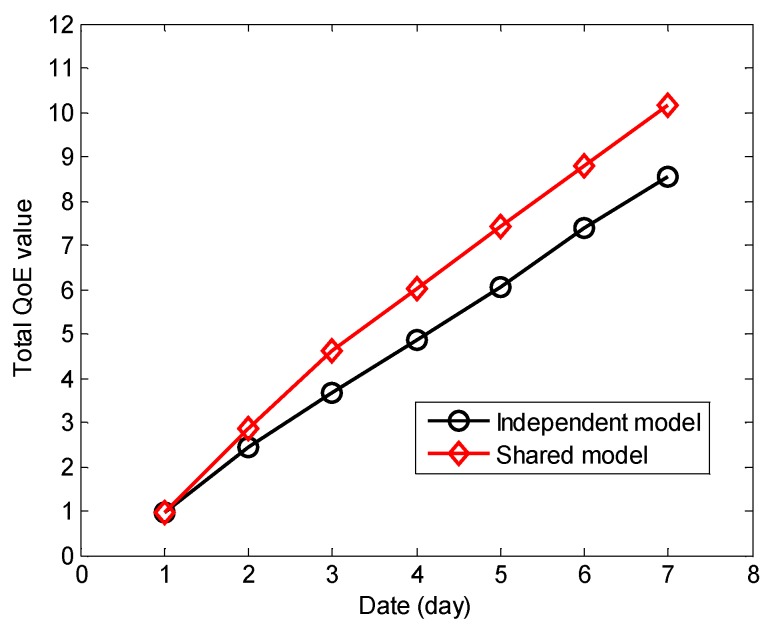
The total QoE value of different model.

Based on the above hypothesis of the relationship between the trigger frequency and the user model, we can see from Figure 14 that the user model with the IM can be confirmed during more than 3 days because the user is not going to all the rooms every day. The user models of all the rooms are independently constructed. However, the user model of the SM can be confirmed in the day 2 because all the user information is shared. Figure 15 shows the total QoE of the different models, and we observe that the total QoE with the SM is higher than the one with the IM.

## 5. Challenges and Opportunities

The AI-SIoT has shown great potentials for future applications, which can enable the intelligent services conveniently and discover new services to meet the ever growing and varying requirements of the users. At the same time, AI-SIoT brings in a lot of business opportunities to the service providers. However, there also exist some challenges.

### 5.1. Personal Information Security Protection

In a SC based on AI-SIoT, people need to share their personal information in the Cloud for intelligent services. The service management layer shall analyze the users’ intentions according to the personal information, predict the needs of users and provide the appropriate services. The resource providers should provide corresponding intelligent services according to the analytical results of the service management layer. Therefore, collecting the users’ personal information is the premise of providing intelligent services. On the other hand, however, sharing the personal data may cause privacy disclosures [58,59,60]. Users’ personal information can be acquired and tampered with by hackers, and the intelligent services can be invaded and destroyed. Therefore, it is a big challenge to protect personal information security under the premise of information sharing.

To ensure the sharing of information in the IoT age, the methods of property/feature encryption can be implemented in the future. Users’ personal sensitive information and their other feature information will be encrypted separately. Because the services are provided according to users’ properties, features and requirements (PFR), services acquired by a certain user can be obtained once again by other people with similar PFR and the personal sensitive information will be not readable for others.

### 5.2. Multidimensional Data Integration

In the SC scenario, the multidimensional user data is relatively complex compared with the simple SH scenario. The multidimensional user data can be integrated and analyzed as a whole [61]. Therefore, the SC scenario can include many intelligent service solutions [62]. It provides great opportunities for resource providers, which satisfy the users’ requirements in a variety of ways and new lifestyles could be stimulate. However, multidimensional data bring in new challenges during the provision of the appropriate service through semantic analysis. In particular, the semantic analysis is based on the semantic matching technology between the intelligent services and the users’ intentions. The multidimensional user data may represent various intentions of users and, correspondingly, there may be various services needed to be scheduled accurately and efficiently. Therefore, how to address concurrent services and how to schedule the service sequence for users are important issues in the developing of the AI-SIoT.

The AI system will solve the most problems in data analysis. The users’ intention, IoT services and multidimensional data can be described by natural language. With the improvement of server performance and the gradual maturity of AI algorithm, AI system can handle a large number of natural language processing tasks in a short period of time. Therefore, the deep analysis and processing of semantic models based on natural language will be the future trend.

## 6. Conclusions

In this paper, we have suggested to grant AI to the SIoT in the SC. We have first discussed the necessity of introducing AI into intelligent services and the key technologies are introduced, including data analysis technologies and semantic technologies. The former is devoted to discovering people’s living patterns. Meanwhile, the latter enable the smart devices to learn the activities and intentions of people. By applying the above mentioned key technologies, we propose the AI-SIoT hybrid service architecture. The AI-SIoT is user-centric and can hide the heterogeneity of different IoT platforms based on various semantic models from the Cloud, including user models, device models and knowledge models. The new type of services can also be modeled and provided to people by the resource providers. We have also provided some use cases of basic intelligent services, semantic annotation, reasoning and service based on associations of semantic models. Finally, we have discussed the opportunities and challenges in commercial and technical fields of the SC. We believe that the AI-SIoT will successfully support SC services in the future.

## Figures and Tables

**Table 1:** Measurement parameters.

Date	Room 1		Room 2		Office 1		Office 2	
	T (°C)	Status	T (°C)	Status	T (°C)	Status	T (°C)	Status
Day 1	35	True	35	False	36	True	34	False
Day 2	35	True	35	True	36	True	35	False
Day 3	33	True	34	True	35	False	34	True
Day 4	35	True	34	False	36	True	34	False
Day 5	36	True	36	False	36	False	35	True
Day 6	35	True	35	False	35	True	35	False
Day 7	35	True	35	True	36	False	35	False

**Table 2:** The QoE of IM and SM in different rooms.

Date	Room 1		Room 2		Office 1		Office 2	
	IM	SM	IM	SM	IM	SM	IM	SM
Day 1	0.47	0.47	Null	Null	0.51	0.51	Null	Null
Day 2	0.47	0.47	0.47	0.68	0.51	0.72	Null	Null
Day 3	0.37	0.54	0.43	0.62	Null	Null	0.43	0.62
Day 4	0.68	0.68	Null	Null	0.51	0.72	Null	Null
Day 5	0.72	0.72	Null	Null	Null	Null	0.47	0.68
Day 6	0.68	0.68	Null	Null	0.68	0.68	Null	Null
Day 7	0.68	0.68	0.47	0.68	Null	Null	Null	Null
